# Safety of Hormonal Contraceptives in Patients With Common Variable Immunodeficiency (CVID) Receiving Immunoglobulin Replacement: Evaluating the Risk of Thromboembolism

**DOI:** 10.7759/cureus.117924

**Published:** 2026-10-14

**Authors:** Lorraine Anderson, Monica Tsai, Ami Thakor Philipp

**Affiliations:** 1 Allergy and Immunology, University of California, Los Angeles, Los Angeles, USA; 2 Allergy and Immunology, University of California Irvine School of Medicine, Irvine, USA

**Keywords:** common variable immunodeficiency, endometriosis, endometriosis and chronic pelvic pain, immunodeficiency, immunoglobulin replacement therapy, intravenous immunoglobulin, subcutaneous immunoglobulin, thromboembolism, use of contraceptives

## Abstract

This case report discusses the use of hormonal contraceptives in patients with common variable immunodeficiency (CVID) who are on immunoglobulin replacement therapy (IgRT) and the risks of thromboembolism.

We present a case of a 32-year-old female with CVID on weekly subcutaneous immunoglobulin (SCIG) who required consultation regarding thromboembolism risk prior to initiation of hormonal contraception for the treatment of dysmenorrhea and endometriosis. In this case, we explore the thromboembolic risks of IgRT and hormonal contraceptives, as well as considerations for an immunodeficient patient on replacement-dose immunoglobulin supplementation with gynecological conditions. While there are no clear guidelines on this issue, treatment considerations involve selection of the appropriate hormonal contraceptive, the appropriate route of contraceptive delivery, and recognition that SCIG replacement has fewer systemic adverse events than intravenous immunoglobulin (IVIG) replacement.

## Introduction

Primary immunodeficiency diseases (PIDDs) are inherited disorders of the immune system that predispose affected individuals to infections, autoimmune disease, immune dysregulation, and malignancies. PIDDs are predominantly humoral (antibody) deficiencies and represent approximately 50% of all undiagnosed primary immunodeficiencies.

Common variable immunodeficiency (CVID) is the most common humoral primary immunodeficiency disorder, with an estimated prevalence/incidence between 1 in 25,000 and 1 in 50,000. Immunoglobulin replacement therapy (IgRT) is the standard treatment for prevention of infectious complications of CVID [1,2].

IgRT is linked to thromboembolic risks due to multiple prothrombotic mechanisms, with hyperviscosity being the most established of these mechanisms. The underlying thromboembolic risk of IgRT can be further increased by patient-specific risk factors. IgRT can be administered as intravenous immunoglobulin (IVIG) or subcutaneous immunoglobulin (SCIG), but IVIG is more frequently associated with an increased risk of thromboembolism [3-5]. Hormonal contraceptives are also associated with an increased risk of thromboembolism [6,7].

There are no evidence-based guidelines on the use of hormonal contraceptives in CVID patients receiving SCIG; thus, this case highlights an important knowledge gap regarding the evaluation of thromboembolic risk specifically with SCIG and hormonal contraception. Consequently, this case highlights that coadministration of IgRT and hormonal contraceptives warrants consideration of the overlapping thromboembolic risks for patients who require both treatments and the evidence utilized in the individualized patient decision-making process.

## Case presentation

A 32-year-old female with CVID presented to the Allergy and Immunology clinic for recommendations regarding the risk of thromboembolism with oral contraceptive (OCP) use and concurrent IgRT. She had a history of severe pelvic pain and irregular and heavy menstrual bleeding. During a sterilization procedure via bilateral laparoscopic salpingectomy, she had an incidental finding of stage 1-2 endometriosis of the pelvis. The proceduralist noted endometrial implants described as powder-burn lesions along the left pelvic sidewall and along the utero-sacral ligaments posteriorly. Images from the procedure showed peritoneal plaques on the uterosacral and ovarian fossa. She was referred to an OBGYN and reported painful menses that were so debilitating that she was almost bedridden during her menstruation. She also reported pain outside of menses, described as crampy abdominal pain associated with nausea, two weeks prior to her menses and lasting two days. The gynecologist diagnosed her with pelvic pain and dysmenorrhea due to endometriosis and advised that she needed ovulatory suppression for management of pain and to prevent worsening of disease.

Two years preceding her endometriosis diagnosis, she was diagnosed by an allergy immunology provider with CVID following a workup of gastrointestinal symptoms, which resulted in a finding of hypogammaglobulinemia. Labs showed low IgG of 341 mg/dL, low IgA at 81 mg/dL, and normal IgM at 59 mg/dL. Lymphocyte enumeration panel showed NK cell lymphopenia, normal levels of T cells and B cells, including (CD27+) memory B cells and switched-memory (IgD-IgM-CD27+) B cells (Table 1). The immune workup was reassuring, with no significant cytopenias. CVID diagnostic criteria were met given the findings of low IgG, low IgA, failed response to pneumococcal vaccine, no evidence of secondary causes of immunodeficiency, such as infection, protein loss, medications, or malignancy, and no evidence of profound T-cell deficiency. She began SCIG replacement due to a history of recurrent bacterial sinopulmonary infections. She reported no reactions to bimonthly SCIG and no history of IVIG use.

**Table 1:** Summary of patient's laboratory data Abbreviations: IgG, Immunoglobulin G; IgA, Immunoglobulin A; IgM, Immunoglobulin M; IgE, Immunoglobulin E; NK, Natural killer cell; mg/dL, milligrams/deciliter; cmm, cells per cubic millimeter; kIU/L, Kilo international units per liter

Parameters	Patient Labs	Reference Range
Immunoglobulins		
IgG	341 (L)	726-1521 mg/dL
IgA	81 (L)	87-426 mg/dL
IgM	59	44-277 mg/dL
IgE	<2	>100 kIU/L
Lymphocyte enumeration		
T-Lymphocytes (CD3+)	76	49-84%
T-Lymphocytes (CD3+)	1599	603-2990 /cmm
T-Helper/Inducer (CD4+)	55	28-63%
T-Helper/Inducer (CD4+)	1119	441-2156 /cmm
T-Suppressor/Cytotoxic (CD8+)	17	10-40%
T-Suppressor/Cytotoxic (CD8+)	344	125-1312 /cmm
Ratio (CD4:CD8)	3.25	0.96-3.93
B-Lymphocytes (CD19+)	20	7-27%
B-Lymphocytes (CD19+)	399	107-698 /cmm
NK (CD56+/CD16+)	3 (L)	4-25%
NK (CD56+/CD16+)	68 (L)	95-640 /cmm
Memory B cells (CD19+CD27+)	26.3	6.3-52.8%
Memory B cells (CD19+CD27+)	104.9	18-145 /cmm
IgM Memory B cells (CD19+CD27+IgM+IgD-)	1.7	0-5.3%
IgM Memory B cells (CD19+CD27+IgM+IgD-)	6.8	0-12 /cmm
Unswitched Memory B cells (CD19+CD27+IgM+IgD+)	12.3	1.7-29.3%
Unswitched Memory B cells (CD19+CD27+IgM+IgD+)	49.1	4-85 /cmm
Switched Memory B cells (CD19+CD27+IgD-IgM-)	9.2	2.3-26.5%
Switched Memory B cells (CD19+CD27+IgD-IgM-)	36.7	7-61 /cmm
Immature B cells (CD19+CD21Low)	5.7	0.2-8.6%
Immature B cells (CD19+CD21Low)	22	0.3-22 /cmm

Her past medical history was notable for irritable bowel syndrome. She was a nonsmoker who seldom drank alcohol and had no personal or family history of hypercoagulability, nor a family history of immunodeficiency. Her gynecologist advised that she consider hormonal contraceptives to address her pelvic pain due to endometriosis. Placement of a progestin-only hormonal intrauterine device (IUD) was painful during insertion, so she requested a combined estrogen and progesterone (E/P) birth control pill. Her gynecologist informed her that combined E/P OCPs have a risk of thromboembolism, and she recalled that her allergy immunology physician also advised her that SCIG has a risk of thromboembolism, so she presented to the Allergy clinic for guidance.

## Discussion

There are risks associated with IgRT based on the route of delivery and how frequently (medication dosing) the medication is given, which can impact the degree of risk. Exact incidences of adverse reactions are difficult to define due to study heterogeneity, differences in patient populations, and IgRT dosing. There are fewer systemic adverse events with SCIG compared to IVIG. Studies show that up to 5% of patients on SCIG experience systemic adverse reactions, which tend to be mild, while local reactions are reported in 75% of patients on SCIG and tend to be transient and mild.

Comparatively, local reactions are uncommon in IVIG patients, while 33% experience systemic adverse reactions during or within 72 hours of the infusion, some of which can be serious and have long-term implications [3,4,8]. Since 2013, IVIG has had a boxed safety warning concerning thromboembolism, which can develop in varied locations, including arteries, veins, and intracranial venous sinuses. Arterial thrombosis is the most common and manifests as myocardial infarctions, pulmonary embolism, and stroke. Arterial events tend to occur within five days after IVIG, while venous events can occur within 1 to 16 days [3,4,8,9].

There are no head-to-head randomized controlled trials comparing thromboembolic risks between the IVIG and SCIG routes of administration. A recent systematic review comparing the thromboembolic safety of SCIG versus IVIG across neurological and immunological indications showed that SCIG is associated with significantly less thromboembolic risk than IVIG (0.14 thromboembolic events/100 patient-years for SCIG versus 1.8 thromboembolic events/100 patient-years for IVIG). Only three thromboembolic events were documented in patients on SCIG, and all patients had prothrombotic risk factors. A key limitation of this meta-analysis is that it included studies in which people were "reporting thromboembolic events in adult patients (≥18 years) receiving IVIg or SCIG for any indication." The IVIG study indications were chronic inflammatory demyelinating polyradiculoneuropathy (CIDP) (44%), myasthenia gravis (MG) (26%), Guillain-Barré syndrome (GBS) (11%), primary immunodeficiency (PID) (11%), and other (8%), while the SCIG study indications were CIDP (57%), PID (29%), and MG (14%). Assuming that only the primary immunodeficiency patients were receiving "replacement dosing of 0.4 g/kg/month" and that CIDP, MG, GBS, and other were receiving "high dose" (generally 2 g/kg/course), then the relative increase in the percentage of high-dose patients receiving IVIG rather than SCIG may account for some of the thromboembolic difference detected in the meta-analysis. Additionally, the total patient-years in the IVIG studies was approximately 10,600, compared to approximately 2,200 in the SCIG studies. These five-fold more patient-years are a critical constraint in the analysis showing the increased events in the IVIG group [9].

Patient-specific risk factors for thrombosis are varied and additive, with studies showing that having multiple risk factors multiplies a patient’s overall thromboembolic risk. In a systematic review and meta-analysis of thrombotic risk in SCIG versus IVIG therapy, the authors highlighted the following patient-specific risk factors for IVIG-related thrombosis: “age over 65, preexisting cardiovascular disease, BMI greater than 30, immobility/bed rest, dehydration, thrombophilia, high-dose IVIG ≥1.5 g/kg/course, and rapid infusion rate” [9]. While SCIG had insufficient thromboembolic event counts to allow for a formal risk factor analysis, thromboembolic events may also occur in patients with no known risk factors following IgRT by any route [3,4,8]. The underlying mechanisms by which IgRT is implicated in thromboembolic events are unclear. However, increased plasma viscosity is proposed by multiple studies to contribute most to the occurrence of arterial and venous thrombosis related to IVIG [3-5,8,9]. While IVIG has a higher risk of thromboembolism as compared to SCIG, Arcani et al. showed that prophylactic systemic anticoagulation did not significantly reduce the IVIG-associated rate of thromboembolism [3]. Interestingly, while SCIG has been used successfully in patients with pre-existing thrombi with immunodeficiency, a review of the medical literature shows a case report of a 20-year-old female CVID patient who developed cerebral venous and sinus thrombosis after using both weekly SCIG with low-dose IgRT and a combined E/P OCP [5,10].

Hormonal contraceptive use is an independent risk factor for thromboembolism. Studies show that combined E/P therapy preparations increase the risk of venous thromboembolism (VTE) by two- to five-fold compared to women who are not pregnant and are not using hormonal contraception (3-15 vs. 1-5 events per 10,000 woman-years) [6]. This thrombogenicity is in part attributable to multiple changes in clotting factors and vasculature, as well as estrogen dose and type of progestin.

In 2024, Skeith and Bates showed that the relative risk (RR) of VTE with combined E/P therapy varies by route of administration and is reported as follows: transdermal patch RR 7.9 (95% CI, 2.5-17.7), vaginal ring RR 6.5 (95% CI, 4.7-8.9), and OCP pill RR 3.5 (95% CI, 2.9-4.3). Among the combined hormonal options, the transdermal patch and vaginal ring showed a higher or similar VTE risk when compared to combined OCPs. Similarly, the RR of VTE with progestin-only contraceptives also varies by route of administration and is as follows: injectable RR 2.7 (95% CI, 1.3-5.5), subdermal implant RR 1.7 (95% CI, 0.6-3.4), intrauterine RR 1.4 (95% CI, 0.2-1.5), and progestin-only pill RR 0.9 (95% CI, 0.6-1.5). Consequently, while progestin-only options have varying VTE risk, they have a lower risk of VTE than combined hormonal options [7]. 

Our patient case is not explicitly expanded upon in the medical literature, as there are no specific studies comparing thromboembolic outcomes between routes of immunoglobulin replacement in CVID patients who require hormonal contraceptives. The above evidence-based literature used in the decision-making for this case includes a heterogeneous mix of randomized controlled studies, prospective and retrospective cohort studies, case series, and case reports reporting on thromboembolic events in adult patients (≥18 years) receiving IVIG or SCIG for various indications. Thus, the main limitation of this case report is the application of data from a heterogeneous evidence base to a single patient with a specific indication for IgRT and a specific need for hormonal contraceptive options, which carries the risk of overinterpreting findings and leading to false causal and management conclusions. In cases such as this, the lack of evidence-based guidelines can lead clinicians to rely on literature searches to help fill in the knowledge gaps, which can produce over-reliance on non-randomized comparisons, indirect extrapolation, and clinical experience. Due to these limitations, close patient follow-up is essential to evaluate for thromboembolism or adverse effects after starting hormonal therapy in patients on IgRT, to help clearly distinguish the evidence in the literature-based estimates from relevant observations in this single patient.

## Conclusions

First-line treatments for endometriosis include hormonal suppression with combined E/P or progestins. There are no established guidelines on the use of hormonal contraceptives in CVID patients on IgRT. Because IgRT for CVID is lifelong, selection of hormonal therapy is one of the few modifiable thrombotic risk factors. Our patient's need for both replacement-dose SCIG and long-term hormonal suppression favored minimizing additional estrogen-related thrombotic risk through selection of a progestin-only contraceptive. In alignment with the findings cited in the medical literature, we recommended progestin-only methods, such as the mini-pill or hormonal IUD, and recommended against injectable and depot progestin subdermal implant options due to the associated risks of thromboembolism. Our patient opted for the progestin-only tablet and tolerated it well while continuing SCIG. At subsequent follow-up three months later, she had no symptoms or findings consistent with thromboembolic disease or adverse events while on concurrent treatment with SCIG and a progestin-only tablet. However, due to the progressive nature of her endometriosis symptoms, she required surgical intervention.

This case highlights the importance of individualized decision-making when encountering knowledge gaps and the absence of evidence-based guidelines. Recommendations regarding the relative safety of specific contraceptive methods should be used cautiously, as this is a single case report with no observed thromboembolic event and limited follow-up. The novelty of evaluating thromboembolic risk specifically with IgRT and hormonal contraception requires further randomized studies to help guide clinical management. Randomized studies can help clinicians better integrate relevant evidence-based data into patient care rather than utilizing heterogeneous studies leading to indirect comparisons, which can lead to overinterpretation of data in the assessment of individual risk stratification of patients with thrombotic risk factors when hormonal therapies are used along with other prothrombotic exposures, such as SCIG. Consequently, for this patient, individualized decision-making with a multidisciplinary approach was integral in optimizing the treatment outcome and minimizing the risk of thromboembolic events. 
